# Health and resource burden of a cancer diagnosis on the caregiver: an analysis of administrative claims data

**DOI:** 10.1186/s12913-021-06938-8

**Published:** 2021-08-30

**Authors:** Lisa M Hess, Siew Hoong Wong-Jacobson, Patrick M Peterson

**Affiliations:** Eli Lilly and Company, Indianapolis, IN 46285 USA

**Keywords:** Health care cost, Resource utilization, Caregiver, Cancer, Outcomes

## Abstract

**Purpose:**

Cancer diagnosis is known to affect the family; however, administrative claims data are not commonly used to evaluate the broader impact of cancer diagnosis. This study was designed to evaluate the feasibility of using claims data to explore the impact of cancer diagnosis on the caregiver.

**Methods:**

IBM Marketscan data were used to identify eligible cancer patients, who were required to have a second adult over the age of 18 (defined as “caregiver” for this study) covered by the same the healthcare policy. Eligible control pairs included any two adults in the same policy with no evidence of cancer; for each pair one adult was randomly assigned to be the “patient control” while their partner was assigned as “caregiver control”. Probabilistic stratified sampling was used select control pairs for analysis by matching the relative frequencies within sex and age group strata to those of patient/caregiver pairs. Eligible control pairs were probabilistically sampled without replacement until the stratum with at least 0.5 % relative frequency had been completely sampled. Caregiver and caregiver control healthcare resource utilization (HCRU), new diagnoses, and healthcare costs were compared during the 12-month post-diagnosis period. Subgroup analyses were conducted by cancer subtypes (breast, colorectal, lung, gastric, sarcoma) and by sex of the patient and caregiver.

**Results:**

A total of 62,893 patient/caregiver pairs and 449,177 control pairs were included. Overall, caregivers used slightly fewer healthcare resources and expended less costs during the 12-month period after the cancer diagnosis than controls (physician visits; 85.8 % vs. 95.7 %; hospitalizations 5.4 % vs. 7.0 %; emergency room visits 15.7 % versus 16.2 %, all *p* ≤ 0.001). This finding was consistent in all subgroup analyses. New diagnoses were lower in the caregiver cohort, except for mental disorders, which were higher than controls (14.3 % vs. 9.9 %, *p* < 0.0001). Psychotherapeutic/antidepressant utilization occurred among 21.0 % of caregivers versus 17.2 % of caregiver controls during this period.

**Conclusions:**

It is feasible to use administrative claims data to evaluate the impact of a cancer diagnosis on the caregiver to evaluate outcomes such as HCRU, diagnoses and costs. These findings raise hypotheses about deferment of health care and increased mental distress during the caregiving period.

## Background

In the United States (U.S.), there are approximately 1,898,160 new cancer diagnoses and 608,570 cancer deaths expected in 2021 alone [1]. Unfortunately, a cancer diagnosis not only affects the person receiving the diagnosis but has an impact on the entire family unit. The burden of a cancer diagnosis to the broader family, and particular to the adult partner or caregiver, is often underrecognized in retrospective observational research, largely due to challenges related to limited real-world data that may be used to quantify the broader impact of a cancer diagnosis.

Caregiving is typically defined as informal support from informal family members whose time and efforts are not covered by insurance. These efforts may include increased financial responsibilities, driving to and from health care appointments, increased responsibilities in the home, such as cleaning and meal preparation, as well as ensuring medication and nutrition intake is maintained. Unlike home health or nursing support, the cost of caregiving is not a reimbursable expense, and individuals caring for a family member with cancer have been documented to suffer loss of employment, reduced productivity, and working extra hours and at lower paying jobs to accommodate the schedule needed to care for a loved one with cancer [2, 3].

Caregivers overall are generally female (65 %) with an average age of 69.4. Only 9 % of caregivers have self-identified as lesbian, gay, bisexual or transgender (LGBT) [4]. While research has quantified the potential costs of time and resources used for informal caregiving [5], few studies have evaluated the impact of caregiving at a population level. What is known about caregivers has been obtained through surveys or qualitative interview data, which have established the range of challenges faced by caregivers. Caregivers of cancer patients have reported anxiety, depression, sleep disturbances, as well as declining quality of life and mental health [6–8]. The evidence related to physical health is less consistent, with only about half of all studies in a review of the literature finding associations between morbidity and caregiving [8].

This study was designed to explore the feasibility of using large administrative claims databases to quantify the impact of cancer diagnosis on an adult caregiver using data resources that allow for large, representative samples of unselected individuals. The goal of this research was to determine if the quantification of caregiver burden associated with cancer may be improved by using large databases for research. This study therefore investigated the hypothesis that health care resource utilization, new diagnoses, and costs would be higher among caregivers compared to matched controls.

## Methods

### Database

This was a retrospective observational study that utilized the IBM (International Business Machines) Health, formerly Truven, MarketScan® databases, which were used under license for the current study. These databases contain de-identified HIPAA-compliant fully integrated patient-level inpatient, outpatient, and drug data from commercial, Medicaid and employer-sponsored Medicare supplemental plans. The databases reflect the real-world healthcare experience of employees, retirees, and dependents covered by the health benefit programs of large employers. The data are collected from approximately 350 different insurance companies and third-party administrators. Marketscan databases have been used in over 300 peer-reviewed articles published in leading journals since 1990. De-identified data are not considered human subjects research according to the U.S. Code of Federal Regulations [9].

### Cohort identification and inclusion criteria

 Family units were identified in the database as two adults over the age of 18 recorded within the same health care policy (one adult was required to be the primary policy holder). To be considered for the cancer cohort, one of the two adults in the family unit was required to have at least two cancer codes reflecting the same anatomical site (e.g. breast, lung, colorectum) on different dates within a 91-day window. The initial cancer diagnosis was required to occur between January 1, 2011 and December 31, 2018. Additionally, the cancer patient was required to have ICD codes for metastatic disease. The second adult in the family unit covered by the health policy was defined as the caregiver. The index date was defined as the first date when a metastatic cancer diagnosis code was observed for the patient. A minimum of 180 days of pre-index continuous enrolment was required for both the cancer patient and the caregiver. Caregivers were further required to have no evidence of cancer within ± 12 months of the index date and to have at least 180 days of post-index continuous enrollment. No a priori sample size was fixed since the intention of the study was to include the maximum number of eligible due to the high number of patients in the Marketscan database.

Control pairs were selected according to a two-step process. First, similar to the cancer cohort, a set of eligible adult control pairs (family units) were identified, with one of each pair randomly assigned as the “patient control” and the other assigned as “caregiver control”. To be eligible, each control pair must have similarly consisted of two adults who were part of the same health care policy. Each control pair was required to have records within the same time period as specified for the patient/caregiver pairs. Each caregiver control was required to have 180 days of continuous enrollment both prior to and following a randomly selected index date. Control pairs were excluded from eligibility if either adult had any evidence of cancer at any time in the database. Second, a probabilistic stratified sampling method was applied to match control pairs to patient/caregiver pairs. Strata were identified among the patient/caregiver cohort based on 4 variables: the sex and age of each of the patient and caregiver. Age in years was categorized into 4 groups (< 40, 40–54, 55–64, or ≥ 65), so that there were potentially up to 64 strata as determined by all possible combinations of age category and sex within pairs. Probabilistic stratified sampling of control pairs was therefore intended to replicate the relative percentages of patient/caregiver pairs between strata, while also maximizing the total quantity of control pairs included in the study. Therefore, the probability of sampling control pairs within any particular stratum was set equal to the relative percentage of patient/caregiver pairs from that stratum, with all eligible control pairs sampled without replacement until the first sufficiently large stratum (those with at least 0.5 % relative frequency) had been completely sampled. This procedure ensured nearly identical relative frequencies of representation between patient/caregiver pairs and control pairs within all large strata, reasonably balanced representation between patient/caregiver pairs and control pairs within smaller strata, while approximately maximizing the sample size of control pairs overall. Index dates for the control pair were randomly selected between January 1, 2011 and December 31, 2018, as no metastases were available to define the index date in the control pair as they were required to have no evidence of cancer. For all cohorts, if there was more than one adult family member holding the same policyholder value, these cases were excluded due to potential data entry errors and lack of ability to clearly define a single ‘caregiver.’ Follow-up data were available through December 31, 2019 at the time of analysis.

### Statistical analysis

The overall goal of the analysis plan was to compare health care resource utilization (HCRU), any new diagnoses, and costs between caregivers versus matched controls (caregiver controls). The study objectives were designed to test the hypothesis that each of these would be higher among caregivers than among caregiver controls.

Baseline demographic characteristics of the patient, patient control, caregiver, and caregiver control were compared using unadjusted comparisons from Student’s t-test for continuous measures and chi-squared test for categorical measures.

HCRU outcomes were compared between the caregiver and caregiver control, including medication utilization, physician visits, emergency room, urgent care, hospitalizations, and surgical procedures. Comparisons of the matched cohorts were conducted using Student’s t-test for continuous measures and Chi-squared test for categorical measures.

New health care diagnoses were evaluated by grouping diagnostic codes consistent with the Current Procedural Terminology (CPT) manual. New diagnoses were those that first appeared on or after the index date, and were compared between the caregiver and caregiver control using Student’s t-test for continuous measures and Chi-squared test for categorical measures.

Costs (both payer and patient out of pocket) were compared between the caregiver and caregiver control using T-test. Additionally, the non-parametric Wilcoxon Rank Sum test was conducted as costs are often not normally distributed, with all costs adjusted to 2018 U.S. dollars using the Medical Care Component of the Consumer Price Index. The primary time of analysis was limited to the 1-year post index period; however, additional analyses were conducted throughout the follow up time period. Due to differential follow up after the initial 1-year period, costs were also evaluated as average monthly costs.

Lastly, while the actual dates of death of cancer patients in this study were not a part of the database, the last activity date in the database might reasonably be assumed to approximate date of death for many of the cancer patients with advanced (metastatic) disease. For those caregivers who remained in a health care plan after the last activity date of the cancer patient, HCRU, new diagnoses, and costs were described to allow evaluation of caregiver outcomes after the possible death of the cancer patient. No imputation was made for missing variables. All analyses were conducted using SAS Enterprise Guide 7.1.

### Subgroup analyses

Due to the heterogeneity of the set of diseases within the broad definition of ‘cancer,’ a series of subgroup analyses were planned *a priori*, and included analyses by primary cancer site (breast, colorectal, gastric, lung cancer, and sarcoma) as well as by sex (male cancer patient, female cancer patient, and by cancer patient-caregiver pairs of the same sex). For each cancer subtype, control pairs were re-selected following the same general procedure as described above for the selection of control pairs generally for the overall study. Within each cancer site, the probability of sampling control pairs within any particular stratum was set equal to the relative percentage of patient/caregiver pairs from that stratum within that cancer subtype, with all eligible control pairs sampled without replacement until the first stratum (those with at least 0.5 % relative frequency) had been completely sampled.

## Results

**Fig. 1:**
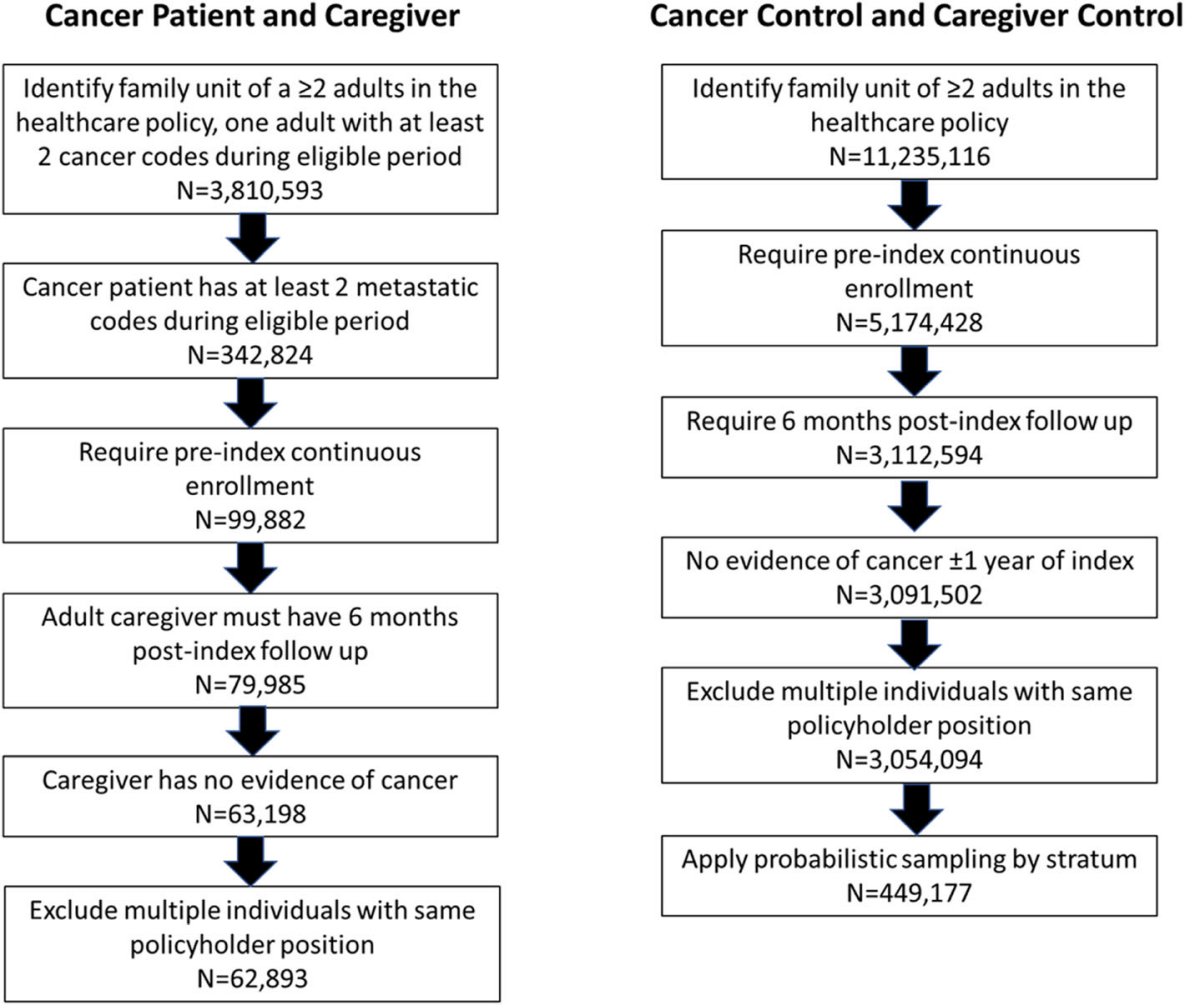
Cohort eligibility diagram. Each N represents a pair of individuals within the same healthcare policy

A total of 62,893 patient/caregiver pairs and 3,054,094 control pairs were eligible for inclusion in this study. The cohort eligibility diagram is presented in Fig. 1.

After applying the selection process there were 449,177 control pairs included in the study. Of the eligible patient/caregiver pairs: 13,174 were included in the breast cancer subgroup; 7,128 in the colorectal cancer subgroup; 1,308 in the gastric cancer subgroup; 9,600 in the lung cancer subgroup; and 907 in the sarcoma subgroup. There were 29,841 in the male patient subgroup, 33,052 in the female patient subgroup, and 458 where the cancer patient and caregiver were the same sex. The results of the selection process for control pairs are summarized in Table 1 and the results by subgroup are summarized in Table 2.

**Table 1:** Baseline characteristics of the patient/patient control and caregiver/caregiver controls after matching

Characteristics at index date	Cancer Patient (*N* = 62,893)	Patient control (*N* = 449,177)	*P*-value	Caregiver (*N* = 62,893)	Caregiver Control (*N* = 449,177)	*P*-value
**Sex, n (%)**						
Female	33,052 (52.6)	235,973 (52.5)	0.93	29,927 (47.6)	213,781 (47.6)	0.96
Male	29,841 (47.4)	213,204 (47.5)		32,966 (52.4)	235,396 (52.4)	
**Age at index, mean (standard deviation, SD) years**	57.7 (11.5)	56.8 (11.8)	< 0.0001	56.9 (11.5)	56.4 (11.6)	< 0.0001
**Age categories, n (%)**						
Age < 40 years	3,873 (6.2)	27,653 (6.2)	1.0	4,136 (6.6)	29,585 (6.6)	1.0
40 ≤ Age < 55	20,006 (31.8)	142,900 (31.8)		21,548 (34.3)	153,882 (34.3)	
55 ≤ Age < 65	24,363 (38.7)	174,112 (38.8)		23,735 (37.7)	169,429 (37.7)	
Age ≥ 65	14,651 (23.3)	104,512 (23.3)		13,474 (21.4)	96,281 (21.4)	
**Health care payer type, n (%)**						
Commercial	48,042 (76.4)	343,924 (76.6)	0.32	49,203 (78.2)	352,032 (78.4)	0.43
Medicare	14,851 (23.6)	105,253 (23.4)		13,690 (21.8)	97,145 (21.6)	
**Health care plan type, n (%)**						
Capitated	7,256 (11.5)	64,165 (14.3)	< 0.0001	7,256 (11.5)	64,237 (14.3)	< 0.0001
Fee for Service	54,242 (86.2)	374,538 (83.4)		54,249 (86.3)	374,462 (83.4)	
Unknown/Missing	1,395 (2.2)	10,474 (2.3)		1,388 (2.2)	10,478 (2.3)	
**US geographic region, n (%)**						
North Central	17,175 (27.3)	124,743 (27.8)	< 0.0001	17,176 (27.3)	124,752 (27.8)	< 0.0001
North East	13,857 (22.0)	79,572 (17.7)		13,861 (22.0)	79,548 (17.7)	
South	21,252 (33.8)	151,522 (33.7)		21,254 (33.8)	151,487 (33.7)	
West	10,120 (16.1)	88,864 (19.8)		10,119 (16.1)	88,876 (19.8)	
Unknown/Missing	489 (0.8)	4,476 (1.0)		483 (0.8)	4,514 (1.0)	
**Employment status of primary policy holder, n (%)**						
Employed	12,346 (19.6)	106,760 (23.8)	< 0.0001	19,104 (30.4)	115,974 (25.8)	0.003
Not Employed	8,659 (13.8)	61,918 (13.8)		9,428 (15.0)	54,941 (12.2)	
Unknown/missing	41,888 (66.6)	280,499 (62.4)		34,361 (54.6)	278,262 (61.9)	
**Charlson Comorbidity Index**^**a**^, **mean (SD)**	2.3 (1.7)	0.4 (1.0)	< 0.0001	0.4 (0.9)	0.4 (1.0)	< 0.0001
**Number of unique medications**^**b**^, **mean (SD)**	7.5 (4.9)	4.6 (4.6)	< 0.0001	4.3 (4.5)	4.5 (4.6)	< 0.0001
**Duration of follow up, mean (SD) months/median (interquartile range, IQR)**	25.4 (20.1)/19.3 (0.1–108.0)	28.3 (20.0)/22.1 (1.7-108.1)	< 0.0001	32.1 (21.9)/25.9 (5.9–108.0)	28.9 (20.5)/22.5 (4.9-108.1)	< 0.0001

^a^Deyo RA, Cherkin DC, Ciol MA. Adapting a clinical comorbidity index for use with ICD-9-CM administrative databases. Journal of clinical epidemiology. 1992 Jun 1;45(6):613-9

^b^Not including systemic cancer therapies; generic drug names were used to identify unique drugs

**Table 2:** Baseline characteristics of caregivers and caregiver controls by subgroup after matching

**Characteristics at index date**	**Breast Cancer Caregiver** (***N* = 13,174)**	**Breast Cancer Caregiver Control** (***N* = 178,966)**	**Colorectal Cancer Caregiver** (***N* = 7,128)**	**Colorectal Cancer Caregiver Control** (***N* = 235,282)**
**Sex, n (%)**				
Female	245 (1.9)	3,293 (1.8)	4,319 (60.6)	142,523 (60.6)
Male	12,929 (98.1)	175,673 (98.2)	2,809 (39.4)	92,759 (39.4)
**Age at index, mean (standard deviation, SD) years**	53.9 (10.7)	53.6 (11.1)	55.8 (11.4)	55.2 (11.6)
**Health care payer type, n (%)**				
Commercial	11,475 (87.1)	155,967 (87.1)	5,806 (81.5)	191,426 (81.4)
Medicare	1,699 (12.9)	22,999 (12.9)	1,322 (18.5)	43,856 (18.6)
**Health care plan type, n (%)**				
Capitated	1,600 (12.1)	25,483 (14.2)	782 (11.0)	33,376 (14.2)
Fee for Service	11,259 (85.5)	149,054 (83.3)	6,197 (86.9)	196,300 (83.4)
Unknown/Missing	315 (2.4)	4,429 (2.5)	149 (2.1)	5,606 (2.4)
**US geographic region, n (%)**				
North Central	3,422 (26.0)	47,198 (26.4)	1,962 (27.5)	64,582 (27.4)
North East	2,644 (20.1)	31,165 (17.4)	1,469 (20.6)	41,528 (17.7)
South	4,616 (35.0)	62,885 (35.1)	2,595 (36.4)	80,100 (34.0)
West	2,402 (18.2)	35,832 (20.0)	1,040 (14.6)	46,581 (19.8)
Unknown/Missing	90 (0.7)	1,886 (1.1)	62 (0.9)	2,491 (1.1)
**Employment status of primary policy holder, n (%)**				
Employed	5,232 (39.7)	64,913 (36.3)	2,033 (28.5)	57,883 (24.6)
Not Employed	1,671 (12.7)	20,771 (11.6)	823 (11.5)	23,952 (10.2)
Unknown/missing	6,271 (47.6)	93,282 (52.1)	4,272 (59.9)	153,447 (65.2)
**Charlson Comorbidity Index**^**a**^, **mean (SD)**	0.3 (0.9)	0.4 (0.9)	0.4 (0.9)	0.4 (0.9)
**Number of unique medications, mean (SD)**	3.5 (4.0)	3.8 (4.2)	4.2 (4.4)	4.5 (4.6)
**Duration of follow up, median (interquartile range, IQR) months**	29.3 (5.9-108.1)	22.1 (4.9-108.1)	25.2 (5.9-107.5)	22.4 (4.9-108.1)
**Characteristics at index date**	**Gastric Cancer Caregiver **(***N* = 1,308)**	**Gastric Cancer Caregiver Control** (***N* = 214,232)**	**Lung Cancer Caregiver **(***N* = 9,600)**	**Lung Cancer Caregiver Control** (***N* = 275,227)**
**Sex, n (%)**				
Female	974 (74.5)	159,880 (74.6)	5,484 (57.1)	157,193 (57.1)
Male	334 (25.5)	54,352 (25.4)	4,116 (42.9)	118,034 (42.9)
**Age at index, mean (SD) years**	57.1 (10.6)	56.6 (11.0)	60.7 (10.4)	60.2 (10.6)
**Health care payer type, n (%)**				
Commercial	1,037 (79.3)	170,402 (79.5)	6,554 (68.3)	189,009 (68.7)
Medicare	271 (20.7)	43,830 (20.5)	3,046 (31.7)	86,218 (31.3)
**Health care plan type, n (%)**				
Capitated	145 (11.1)	30,865 (14.4)	944 (9.8)	39,404 (14.3)
Fee for Service	1,130 (86.4)	178,315 (83.2)	8,457 (88.1)	229,759 (83.5)
Unknown/Missing	33 (2.5)	5,052 (2.4)	199 (2.1)	6,064 (2.2)
**US geographic region, n (%)**				
North Central	362 (27.7)	59,756 (27.9)	3,029 (31.6)	80,068 (29.1)
North East	312 (23.9)	38,081 (17.8)	2,140 (22.3)	49,598 (18.0)
South	431 (33.0)	71,763 (33.5)	3,173 (33.1)	88,974 (32.3)
West	195 (14.9)	42,400 (19.8)	1,172 (12.2)	54,024 (19.6)
Unknown/Missing	8 (0.6)	2,232 (1.0)	86 (0.9)	2,563 (0.9)
**Employment status of primary policy holder, n (%)**				
Employed	387 (29.6)	47,195 (22.0)	2,575 (26.8)	56,683 (20.6)
Not Employed	188 (14.4)	20,564 (9.6)	2,146 (22.4)	44,434 (16.1)
Unknown/missing	733 (56.0)	146,473 (68.4)	4,879 (50.8)	174,110 (63.3)
**Charlson Comorbidity Index**^**a**^, **mean (SD)**	0.4 (1.0)	0.4 (0.9)	0.5 (1.0)	0.5 (1.1)
**Number of unique medications, mean (SD)**	4.7 (4.9)	4.7 (4.7)	5.0 (4.8)	5.0 (4.8)
**Duration of follow up, median (IQR) days**	21.2 (6.0-107.5)	22.7 (5.9-108.1)	22.7 (5.9-106.8)	22.7 (5.9–108.0)
**Characteristics at index date**	**Sarcoma Caregiver **(***N* = 907**)	**Sarcoma Caregiver Control** (***N* = 142,992**)	**Male Cancer Patient Caregiver **(***N* = 29,841)**	**Male Cancer Patient Caregiver Control **(***N* = 213,204**)
**Sex, n (%)**				
Female	507 (55.9)	79,985 (55.9)	29,655 (99.4)	211,871 (99.4)
Male	400 (44.1)	63,007 (44.1)	186 (0.6)	1,333 (0.6)
**Age at index, mean (SD) years**	53.9 (11.9)	53.6 (12.0)	57.4 (11.3)	56.8 (11.4)
**Health care payer type, n (%)**				
Commercial	756 (83.4)	119,728 (83.7)	22,960 (76.9)	164,126 (77.0)
Medicare	151 (16.6)	23,264 (16.3)	6,881 (23.1)	49,078 (23.0)
**Health care plan type, n (%)**				
Capitated	86 (9.5)	20,439 (14.3)	3,411 (11.4)	30,755 (14.4)
Fee for Service	804 (88.6)	119,208 (83.4)	25,760 (86.3)	177,594 (83.3)
Unknown/Missing	17 (1.9)	3,345 (2.3)	670 (2.2)	4,855 (2.3)
**US geographic region, n (%)**				
North Central	224 (24.7)	38,667 (27.0)	8,130 (27.2)	60,258 (28.3)
North East	189 (20.8)	25,078 (17.5)	6,753 (22.6)	38,003 (17.8)
South	312 (34.4)	49,362 (34.5)	10,164 (34.1)	70,704 (33.2)
West	173 (19.1)	28,429 (19.9)	4,564 (15.3)	42,075 (19.7)
Unknown/Missing	9 (1.0)	1,456 (1.0)	230 (0.8)	2,164 (1.0)
**Employment status of primary policy holder, n (%)**				
Employed	297 (32.7)	37,685 (26.4)	6,912 (23.2)	38,304 (18.0)
Not Employed	100 (11.0)	13,557 (9.5)	3,033 (10.2)	15,308 (7.2)
Unknown/missing	510 (56.2)	91,750 (64.2)	19,896 (66.7)	159,592 (74.9)
**Charlson Comorbidity Index**^**a**^, **mean (SD)**	0.3 (0.7)	0.4 (0.9)	0.4 (0.9)	0.4 (0.9)
**Number of unique medications, mean (SD)**	4.1 (4.2)	4.3 (4.5)	4.9 (4.8)	4.9 (4.8)
**Duration of follow up, median (IQR) months**	23.0 (6.0-103.4)	22.3 (5.9–108.0)	23.4 (5.9-107.9)	22.8 (5.9–108.0)
**Characteristics at index date**	**Female Cancer Patient Caregiver **(***N* = 33,052**)	**Female Cancer Patient Caregiver Control **(***N* = 235,973**)	**Same-sex Caregiver **(***N* = 458**)	**Same-sex Caregiver Control **(***N* = 3,243**)
**Sex, n (%)**				
Female	272 (0.8)	1,910 (0.8)	272 (59.4)	1,910 (58.9)
Male	32,780 (99.2)	234,063 (99.2)	186 (40.6)	1,333 (41.1)
**Age at index, mean (SD) years**	56.5 (11.6)	56.1 (11.8)	52.0 (9.6)	51.7 (9.9)
**Health care payer type, n (%)**				
Commercial	26,243 (79.4)	187,906 (79.6)	423 (92.4)	2,994 (92.3)
Medicare	6,809 (20.6)	48,067 (20.4)	35 (7.6)	249 (7.7)
**Health care plan type, n (%)**				
Capitated	3,845 (11.6)	33,482 (14.2)	116 (25.3)	817 (25.2)
Fee for Service	28,489 (86.2)	196,868 (83.4)	337 (73.6)	2,381 (73.4)
Unknown/Missing	718 (2.2)	5,623 (2.4)	5 (1.1)	45 (1.4)
**US geographic region, n (%)**				
North Central	9,046 (27.4)	64,494 (27.3)	59 (12.9)	530 (16.3)
North East	7,108 (21.5)	41,545 (17.6)	108 (23.6)	578 (17.8)
South	11,090 (33.6)	80,783 (34.2)	146 (31.9)	994 (30.7)
West	5,555 (16.8)	46,801 (19.8)	124 (27.1)	1,010 (31.1)
Unknown/Missing	253 (0.8)	2,350 (1.0)	21 (4.6)	131 (4.0)
**Employment status of primary policy holder, n (%)**				
Employed	12,192 (36.9)	77,670 (32.9)	194 (42.4)	1,129 (34.8)
Not Employed	6,395 (19.3)	39,633 (16.8)	50 (10.9)	214 (6.6)
Unknown/missing	14,465 (43.8)	118,670 (50.3)	214 (46.7)	1,900 (58.6)
**Charlson Comorbidity Index**^**a**^, **mean (SD)**	0.4 (1.0)	0.5 (1.0)	0.7 (1.7)	0.6 (1.5)
**Number of unique medications, mean (SD)**	3.9 (4.2)	4.1 (4.3)	4.6 (4.8)	4.8 (4.8)
**Duration of follow up, median (IQR) months**	28.1 (5.9–108.0)	22.1 (4.9-108.1)	26.0 (6.1-106.4)	21.6 (6.0-108.0)

^a^Deyo RA, Cherkin DC, Ciol MA. Adapting a clinical comorbidity index for use with ICD-9-CM administrative databases. Journal of clinical epidemiology. 1992 Jun 1;45(6):613-9

### Health care resource utilization and costs

The most common medications used by caregivers and by caregiver controls during the 6-month pre-index period as well as during the 12-month post index period are summarized in Table 3.

**Table 3:** Most commonly used medications (by class) among caregivers and caregiver controls during the 6- month pre-index and 12-month post-index period, respectively^a^

Medications	Six-month pre-index period		12-month post index period	
	**Caregiver** (***n* = 62,893)**	**Caregiver control **(***n* = 499,177**)	**Caregiver** (***n* = 62,893)**	**Caregiver control** (***n* = 499,177)**
**Total number of unique medications, mean (standard deviation, SD)**^**b**^	4.3 (4.5)	4.5 (4.6)	6.1 (5.8)	6.3 (5.8)
**Drug class, n (%)**				
Antihyperlipidemic drugs, not elsewhere classified (NEC)	17,784 (28.3)	126,906 (28.3)	19,891 (31.6)	143,847 (32.0)
Psychotherapeutics, antidepressants	9,758 (15.5)	65,620 (14.6)	13,188 (21.0)	77,160 (17.2)
Cardiac, beta blockers	9,615 (15.3)	67,265 (15.0)	10,852 (17.3)	75,821 (16.9)
Cardiac, ACE inhibitors	9,429 (15.0)	70,892 (15.8)	10,731 (17.1)	79,487 (17.7)
Cardiac, calcium channel blockers	6,817 (10.8)	48,666 (10.8)	7,781 (12.4)	55,853 (12.4)
Cardiac, NEC	6,790 (10.8)	46,939 (10.5)	7,620 (12.1)	53,744 (12.0)
Analgesic/antipyretic, opiate agonists	8,223 (13.1)	68,557 (15.3)	11,634 (18.5)	96,325 (21.4)
Analgesics/antipyretic, nonsteroidal anti-inflammatory agents (NSAIDs)	7,472 (11.9)	60,674 (13.5)	11,044 (17.6)	85,624 (19.1)
Gastrointestinal drug miscellaneous, NEC	7,329 (11.7)	51,266 (11.4)	9,105 (14.5)	62,922 (14.0)
Adrenals & comb, NEC	6,565 (10.4)	49,119 (10.9)	10,084 (16.0)	75,527 (16.8)
Thyroid/anti-thyroid, thyroid/hormones	6,127 (9.7)	42,104 (9.4)	6,639 (10.6)	46,493 (10.4)
Antibiotics, penicillins	6,256 (9.9)	43,294 (9.6)	10,539 (16.8)	77,020 (17.1)
Antidiabetic agents, miscellaneous	5,932 (9.4)	44,211 (9.8)	6,919 (11.0)	50,748 (113.3)
Antibiotics, erythromycin	4,851 (7.7)	35,126 (7.8)	8,356 (13.3)	60,798 (13.5)
Anxiolytics, sedatives and hypnotics, benzodiazepines	4,811 (7.6)	30,820 (6.9)	8,094 (12.9)	40,611 (9.0)
Anti-Inflammatory agents, eye, ears, nose, & throat (EENT), NEC	4,719 (7.5)	35,335 (7.9)	7,011 (11.1)	53,702 (12.0)

^a^All other classes of drugs were used by < 10 % of patients in each cohort

^b^*p*<0.0001 during both the pre-index and post-index periods, Student’s t-test. Unique medications identified by generic drug name

During the pre-index period, caregivers and caregiver controls had similar medication use (all within 1–2 % points). During the 12-month post-index period, slight numeric differences were observed, with psychotherapeutics/antidepressants utilized among 21.0 % of caregivers versus 17.2 % of caregiver controls, and benzodiazepines used among 12.9 % of caregivers versus 9.0 % of caregiver controls. Differences in medication utilization were most pronounced in the gender subgroups, with 28.9 % of caregivers of male patients using psychotherapeutics/antidepressants during the 12-month post index period versus 23.0 % of caregiver controls to male patient controls. For caregivers and caregiver controls of female cancer patients/controls, these drugs were used by 13.8 and 11.9 % during the 12-month post-index period. Among same-sex patient/caregiver and control pairs, utilization was nearly identical, with 27.5 % of caregivers versus 27.0 % of caregiver controls receiving psychotherapeutics/antidepressants during the 12-month post-index period.

**Fig. 2:**
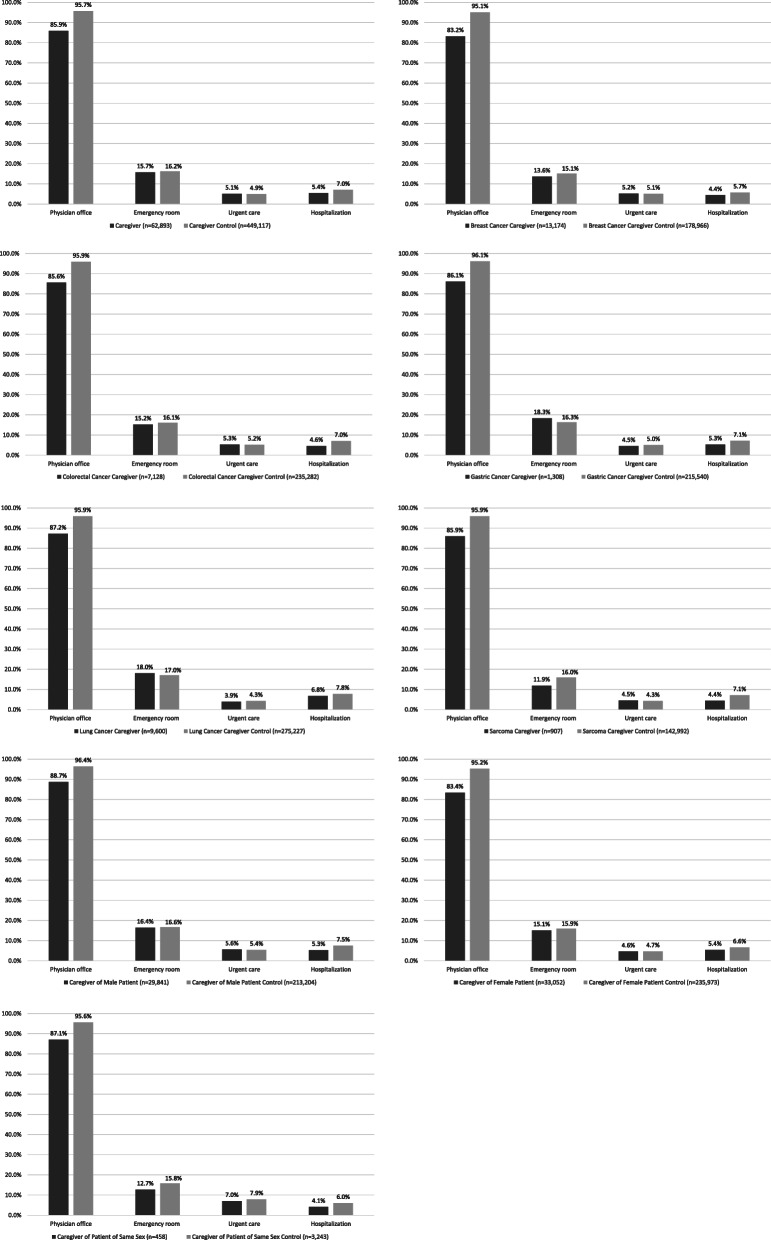
Proportion utilizing health care resources at least once during the 12-month post-index period

There were slightly fewer health care encounters among caregivers versus controls during the 12-month post-index period. Physician office visits occurred among 85.9 % of caregivers versus 95.7 % of controls (*p* < 0.0001), hospitalizations occurred among 5.4 % of caregivers versus 7.0 % of controls (*p* < 0.0001), emergency room visits were observed among 15.7 % of caregivers versus 16.2 % of controls (*p* ≤ 0.001), but there were no significant differences in urgent care visits (5.1 % versus 4.9 %, *p* = 0.23). These findings were consistent across all subgroups in this study (Fig. 2). The lower HCRU was consistent with lower health care costs recorded among caregivers than controls during this 12-month time period. The average total patient out of pocket costs for caregivers was $519.10 (SD=$1,446.60) and for caregiver controls was $723.10 (SD=$1,446.70) *p* < 0.0001. The average total payer costs for caregivers were $3,835.20 (SD=$18,790.30) and for caregiver controls was $4,452.80 (SD=$17,249.90), *p* < 0.0001.

### New diagnoses

**Fig. 3:**
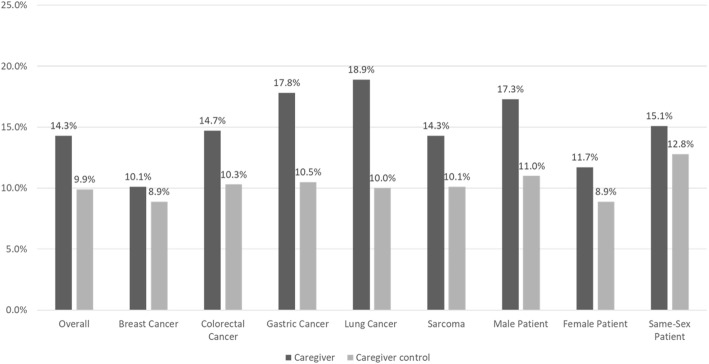
Proportion with new mental disorder diagnoses during the 12-month post-index period

There were statistically significant differences in several new diagnoses between caregivers and caregiver controls, with caregivers having a greater number of new diagnoses in the code range for mental disorders versus controls (14.3 % versus 9.9 %, *p* < 0.0001). All other categories of diagnoses did not vary more than 1–2 % between caregivers and controls. The raw difference in new mental disorder diagnoses varied by subgroup, but all followed a similar pattern with caregivers having a greater proportion of new mental disorder diagnoses; all *p* < 0.0001 versus caregiver controls other than the same-sex patient subgroup, which was not statistically significant (Fig. 3).

### Caregiver outcomes after the cancer patient death

**Fig. 4:**
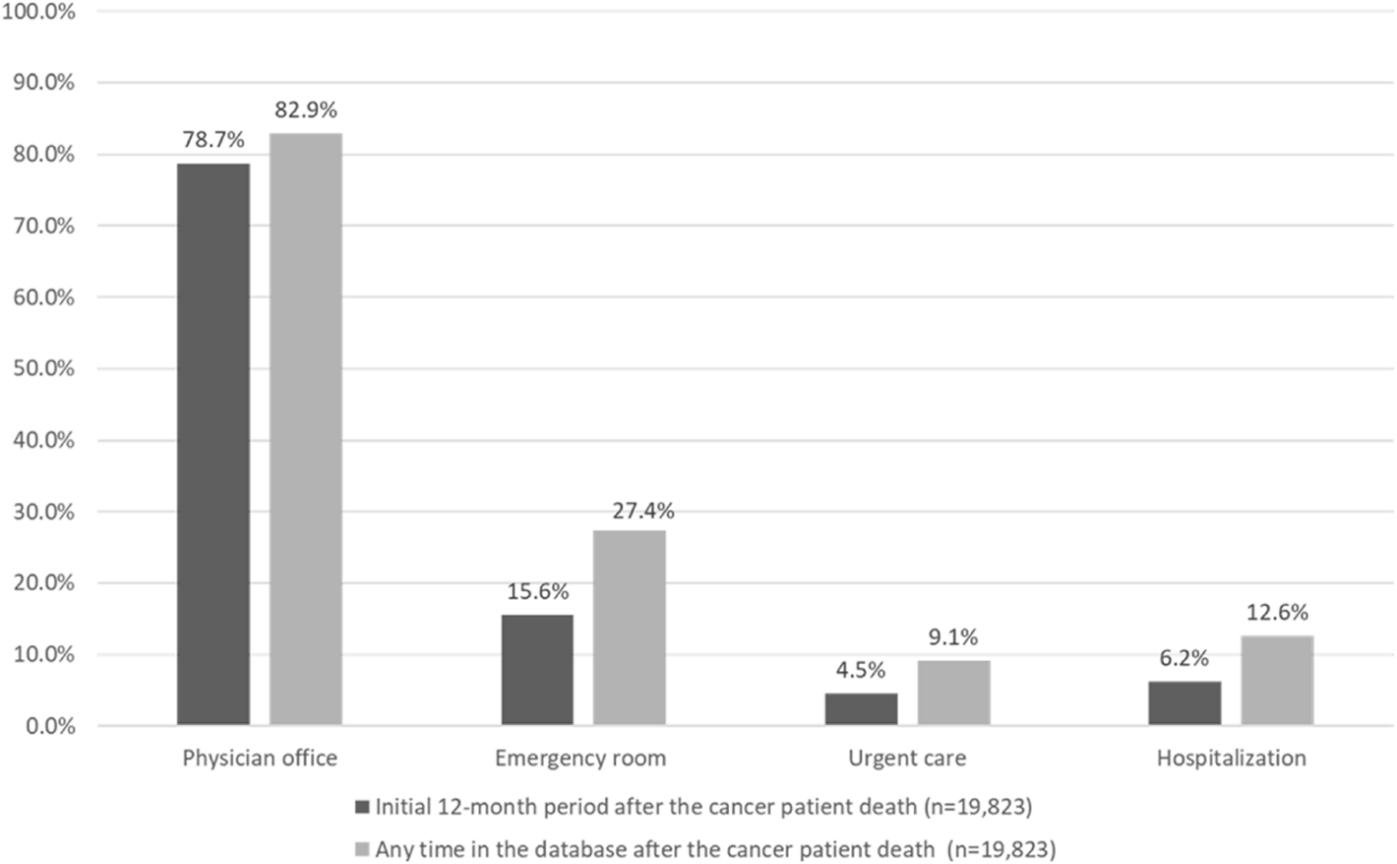
Healthcare resource utilization among caregivers after the death^a^ of the cancer patient (*n* = 19,823). ^a^ Death was assumed based on the last activity date of the cancer patient in the database

Of all the caregivers, 19,823 remained in the health plan after the cancer patient death (estimated based on last activity date of the cancer patient). The characteristics of this caregiver group was similar to the overall caregiver cohort; 49.3 % were female and surviving caregivers had a mean age of 59.2 (SD = 11.1) (Table 4). The median duration of follow-up after the estimated death of the cancer patient was 18.1 months (interquartile range, IQR = 7.0-36.1). Healthcare resource utilization during this period is summarized in Fig. 4.

**Table 4:** Characteristics of caregivers remaining in health plans after the death^a^ of the cancer patient

Characteristics at index date	Caregivers (*N* = 19,823)
**Sex, n (%)**^**b**^	
Female	9,776 (49.3)
Male	10,047 (50.7)
**Age at index, mean (standard deviation, SD) years**	59.2 (11.1)
**Health care payer type, n (%)**	
Commercial	14,693 (74.1)
Medicare	5,130 (25.9)
**Health care plan type, n (%)**	
Capitated	2,134 (10.8)
Fee For Service	17,263 (87.1)
Unknown/Missing	426 (2.1)
**US geographic region, n (%)**	
North Central	5,891 (29.7)
North East	4,080 (20.6)
South	6,993 (35.3)
West	2,734 (13.8)
Unknown/Missing	125 (0.6)
**Employment status of primary policy holder, n (%)**	
Employed	8,373 (42.2)
Not Employed	5,503 (27.8)
Unknown/missing	5,947 (30.0)
**Charlson Comorbidity Index, mean (SD)**	0.4 (1.0)
**Number of unique medications**^**d**^, **mean (SD)**	4.7 (4.6)

^a^Death was assumed based on the last observation of the cancer patient in the database

^b^143 caregivers in this cohort cared for a cancer patient of the same sex

^b^Deyo RA, Cherkin DC, Ciol MA. Adapting a clinical comorbidity index for use with ICD-9-CM administrative databases. Journal of clinical epidemiology. 1992 Jun 1;45(6):613-9

During the initial 12-month period after the death of the cancer patient, physician office visits were observed among 78.7 % and emergency room visits occurred among 15.6 %. New diagnoses are summarized in Table 5 for the overall caregiver cohort both during the caregiving period as well as after the death of the cancer patient.

**Table 5:** New healthcare diagnoses among caregivers during the 12-month post-index period and after the death of the cancer patient

Diagnostic Category^a^	During the 12-month post-index period (*n* = 62,893)	During the initial 12-month period after the death of the cancer patient (*n* = 19,823)	Any time after the death of the cancer patient (*n* = 19,823)
	**n (%)**	**n (%)**	**n (%)**
Infectious and Parasitic Diseases	5,303 (8.4)	1,828 (9.2)	3,641 (18.4)
Endocrine, Nutritional and Metabolic Diseases, Immunity Diseases	12,463 (19.8)	4,069 (20.5)	5,536 (27.9)
Diseases of the Blood and Blood-Forming Organs	3,557 (5.7)	1,326 (6.7)	2,655 (13.4)
Mental Disorders	9,023 (14.3)	3,891 (19.6)	5,459 (27.5)
Diseases of the Nervous System and Sense Organs	12,270 (19.5)	4,013 (20.2)	6,037 (30.5)
Diseases of the Circulatory System	10,563 (16.8)	3,575 (18.0)	4,940 (24.9)
Diseases of the Respiratory System	12,230 (19.4)	3,654 (18.4)	5,830 (29.4)
Diseases of the Digestive System	10,196 (16.2)	3,301 (16.7)	5,653 (28.5)
Diseases of the Genitourinary System	9,893 (15.7)	3,363 (17.0)	5,550 (28.0)
Complications of Pregnancy, Childbirth, and the Puerperium	134 (0.2)	15 (0.1)	35 (0.2)
Diseases of the Skin & Subcutaneous Tissue	9,480 (15.1)	3,215 (16.2)	5,529 (27.9)
Diseases of the Musculoskeletal System and Connective Tissue	13,376 (21.3)	4,331 (21.8)	6,423 (32.4)
Certain Conditions Originating in the Perinatal Period	38 (0.1)	6 (0.0)	23 (0.1)
Symptoms, Signs, and Ill-defined Conditions	17,281 (27.5)	5,524 (27.9)	7,420 (37.4)
Injury and Poisoning	8,397 (13.4)	2,735 (13.8)	5,024 (25.3)
Supplementary Classification of External Causes of Injury and Poisoning	1,950 (3.1)	715 (3.6)	1,688 (8.5)

^a^based on general ICD-9-CM and ICD-10-CM categories

The most common diagnoses during the 12-month post-death period observed were symptoms, signs, and abnormal clinical and laboratory findings (27.9 %), diseases of the musculoskeletal system and connective tissue (21.8 %), endocrine, nutritional/metabolic and immunity disorders (20.5 %), and diseases of the nervous system and sense organs (20.2 %).

## Discussion

 This study examined the ability of administrative claims data to be used to examine the impact of a cancer diagnosis on caregivers (defined as adult co-policy holders of the cancer patient in this study). While the amount of time invested in informal caregiving is not recorded in claims databases, the findings from this study suggest that during the year following diagnosis, adult caregivers may forego health care for themselves as their focus is on the health and wellbeing of the cancer patient. This was demonstrated by the consistent lower rate of health care resources and costs expended versus a matched control cohort overall as well as across tumor site- and sex-specific subgroup analyses.

Despite less frequent health care encounters, caregivers had significantly greater diagnoses in the range of mental disorders during this time period. These findings are consistent with prior published literature that have reported caregiver anxiety, depression, and declining mental health [6–8]. In the current study, the observed differences were largest among caregivers of male patients, most of whom were female caregivers. This pattern was also observed among cancers that were more often diagnosed among men, such as lung cancer. The only caregiver subgroup that did not show any significant difference in mental disorders versus controls was among the same sex cancer patient/caregiver pairs. This may in part be due to the small sample size that limits the ability to detect differences, or simply due to the true lower frequency of diagnoses among caregivers during this one-year period. Same-sex caregivers did not appear to have different rates of healthcare encounters (e.g. physician visits) than the other caregiver groups during the observation period. The exact diagnoses observed were not evaluated in this study, but warrants further investigation. The diagnoses within the range of mental disorders and specific medications prescribed should be explored in future study to better understand what is occurring.

Due to limited variables in the database, some assumptions had to be made when interpreting the variables in this dataset. There are reasons why the cancer patient may no longer be covered by insurance after diagnosis for reasons other than death. It is possible the cancer patient discontinued their health care plan to receive Medicare without continuing the commercial supplement, while other household members stayed on the commercial plan. If this were the case, the cancer patient may no longer be observed in the database and could have incorrectly been assumed to have died. In this study, the assumption was that most people with cancer who were no longer observed in the database while the caregiver partner continued to have claims submitted would be due to death, but no data were available to further clarify if a death had occurred. Therefore, the cohort of patients followed after the death of the cancer patient could have included some individuals who were continuing to be caregivers for the cancer patient but whose care was no longer being recorded in claims.

Additionally, the relationship of the adult caregiver to the patient is unknown. It would be expected that for a health care policy to be shared among adults that most of these individuals would be spouses or domestic partners; however, adult children could have been included in the policy. In the case of multiple adults within the same policy, the caregiver was selected as policy holder 2, which is typically the spouse/partner. However, an adult child could have been policy holder 2 in the case of a single-parent household with coverage through the Affordable Care Act, which extended health care coverage through age 26. While the assumption was made that all caregivers are likely spouses or adults in domestic partnerships, and the age distribution of caregivers suggests this assumption was not incorrect, the nature of the cancer patient-caregiver relationships could not be verified in this database. While the risk of including a child age 18–26 was low due to the higher age of onset of metastatic cancer diagnoses as observed in this study, future research of diseases more common in younger adults may wish to exclude or further evaluate the cases that include a partner who is younger than 27 years of age to determine the risk of inclusion of a child-parent relationship.

These data also do not contain information to verify actual caregiving activities. There may have been other formal or informal caregivers who performed these activities for the individual diagnosed with cancer. Therefore, the caregiver in this study can only be verified as an adult member of the household. Attributing a caregiver role to this individual assumes that some responsibilities were taken for the care of the patient during this time, but also is not verifiable in this database.

The strengths of a large database provide more representative and generalizable data about the impact of a metastatic cancer diagnosis on adult caregiver family members than previously published. This study suggests that even with the limitations of the variables collected, this can be investigated. However, the strength of a large dataset also leads to many significant findings that may not have meaningful values simply due to the power of a large dataset to detect very small differences. In this study, we did not report all significant findings, but those that were also associated with a difference in rates or point estimates that are may be large enough to represent meaningful differences between groups to avoid overstating the role of statistical significance.

## Conclusions

It is feasible to use administrative claims data to evaluate the impact caring for a patient with a metastatic cancer diagnosis. These findings raise hypotheses about the potential deferment of health care during the caregiving period, and the increased distress of this time as observed by mental disorders diagnosed and medications utilized. This study establishes the strengths of claims data to further investigate the challenges of caregiving to provide data that can inform the development of novel solutions to care for the caregiver during a time when their own wellbeing may be neglected.

## Data Availability

The data that support the findings of this study are available from IBM but restrictions apply to the availability of these data, which were used under license for the current study, and so are not publicly available.

## Abbreviations

CPT: Current Procedural Terminology
HCRU: Health care resource utilization
IBM: International Business Machines
LGBT: Lesbian, gay, bisexual or transgender
SD: Standard deviation
